# Endovascular Management of Spontaneous Subclavian Artery Dissection

**DOI:** 10.7759/cureus.39828

**Published:** 2023-06-01

**Authors:** Sibasankar Dalai, Aravind V Datla, Suresh K Korada, Sailesh Modi, Hemanth K Bura

**Affiliations:** 1 Interventional Neuroradiology, Medicover Hospitals, Visakhapatnam, IND; 2 Internal Medicine, Medicover Hospitals, Visakhapatnam, IND; 3 Neurology, Medicover Hospitals, Visakhapatnam, IND; 4 Neurology, Queens NRI Hospital, Visakhapatnam, IND; 5 Emergency Medicine, Medicover Hospitals, Visakhapatnam, IND

**Keywords:** endovascular interventions, acute upper limb ischemia, catheter-directed thrombolysis, endovascular stenting, subclavian artery dissection

## Abstract

Spontaneous subclavian artery dissection (SCAD) is a rare clinical observation with very few cases reported in the medical literature. We describe a rare case of a 50-year-old female patient who presented with symptoms of critical limb ischemia of the right upper extremity. A digital subtraction angiogram (DSA) revealed a dissection in the proximal course of the subclavian artery (SCA). Prompt recanalization with endovascular therapy produced an excellent result.

## Introduction

Isolated subclavian artery dissection (SCAD) is a rare occurrence, usually associated with arterial catheterization, trauma, connective tissue disorders, or anomalies of the aortic arch and other great vessels [1,2]. Only 10 cases have been reported in the medical literature with no antecedent cause, and the majority of them were managed conservatively [3]. In most cases, a good clinical outcome was achieved through conservative management with anticoagulation, antiplatelets, or a combination of both, along with aggressive blood pressure control to prevent the extension of the dissection [2,3]. We report a case of spontaneous SCAD with no contributing factors, leading to critical limb ischemia of the right upper extremity, managed with endovascular therapy. Early recognition and timely intervention produced an exceptional result.

## Case presentation

**Figure 1:**
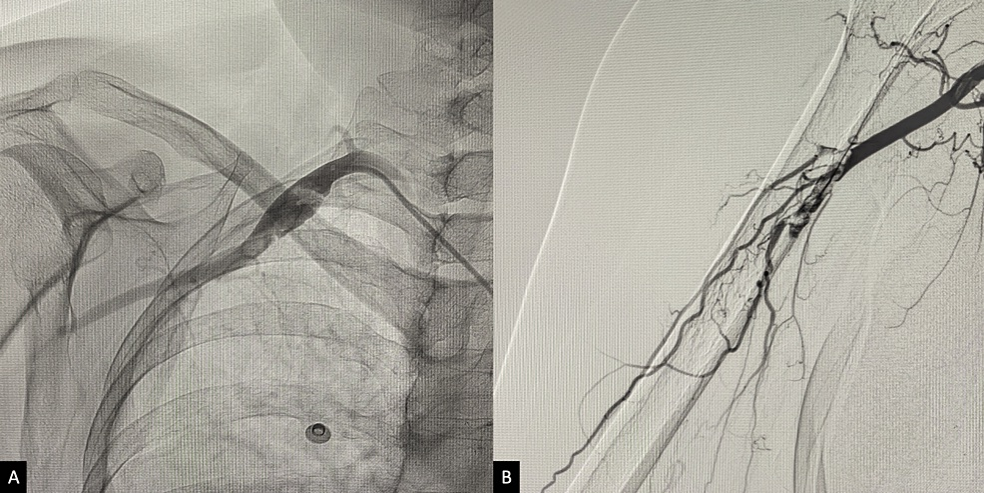
Initial DSA of the right upper limb demonstrating thrombosis of the SCA and partial filling of the axillary artery (A), with no flow beyond the proximal course of the brachial artery (B). DSA, digital subtraction angiogram; SCA, subclavian artery.

A 50-year-old female patient presented to the emergency department with a sudden onset of severe pain in her right upper limb and discoloration of the fingers. During the examination, it was found that she had minimal paresthesia in the right forearm and hand, along with mild weakness in the right upper limb, which corresponds to Rutherford Classification 2b [4]. The patient had no medical comorbidities, did not smoke, and did not consume alcohol or recreational drugs. She did not have a history of trauma or sports activity. During the initial physical examination, her vitals were stable, except for mild tachycardia. The radial pulse was absent, and the limb was cold and pale. The arterial Doppler revealed an acute thrombotic occlusion of the right subclavian artery (SCA), with no flow to the distal portion of the limb. Anticoagulation was initiated (5000 units of intravenous heparin), and a digital subtraction angiogram (DSA) of the right upper limb was performed via the right transfemoral arterial route. The DSA revealed an occlusion of the subclavian artery with partial filling of the axillary artery and no flow beyond the distal end in the right upper extremity (Figure 1). A decision was made to proceed with endovascular revascularization of the right upper limb.

Procedure

**Figure 2:**
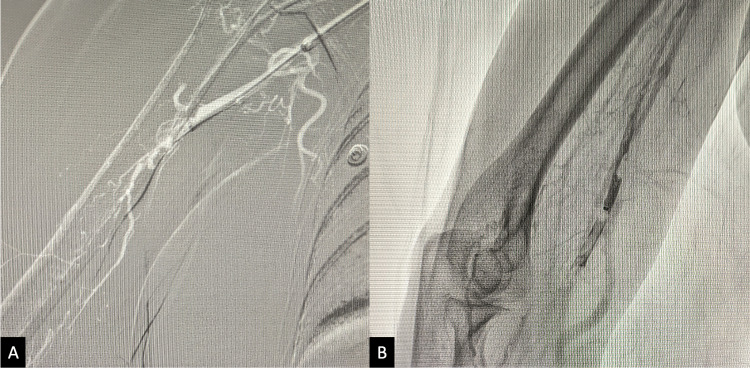
Intraprocedural fluoroscopy showing the navigation of the guide-wire beyond the thrombotic occlusion of the axillary artery (A), and thromboaspiration (B).

A 7F long sheath (Destination^TM^, Terumo Interventional Systems, Somerset, New Jersey, United States) was inserted from the right transfemoral arterial route into the proximal part of the right SCA. A 6.4F distal access catheter (DAC) (Stryker Corporation, Kalamazoo, Michigan, United States) was introduced into the thrombotic occlusion of the SCA, all the way into the brachial artery (BA). Thromboaspiration was performed with partial recanalization of the SCA, BA, and the proximal parts of the radial and ulnar arteries (Figure 2).

**Figure 3:**
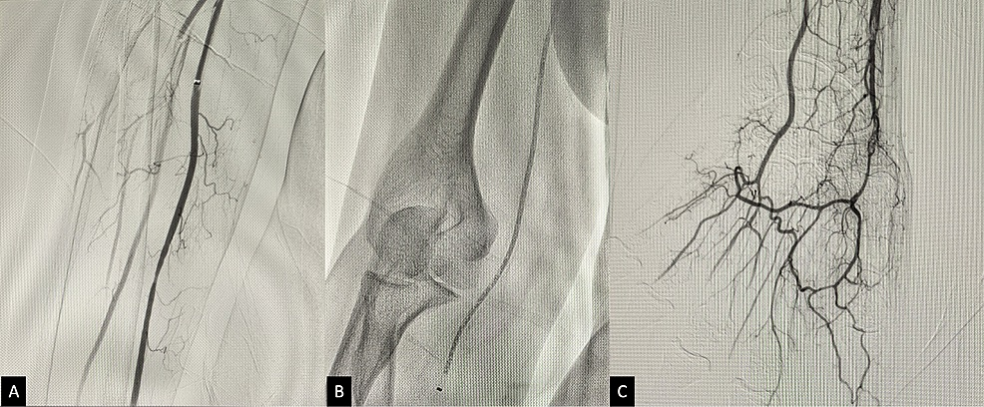
After thrombolysis, DSA was performed, which showed improved revascularization (A). Further thromboaspiration (B) was carried out until adequate recanalization of the distal limb was achieved, up to the level of the palmar arch (C). DSA, digital subtraction angiogram.

The 7F long sheath and DAC were exchanged for an 8F Introducer sheath (Cordis Corporation, Hialeah, Florida, United States), a diagnostic 4F catheter (SIM2, Cordis Corporation) was placed within the proximal half of the SCA that was having residual thrombus, and catheter-directed thrombolysis was initiated. Alteplase (20 mg in 50 ml of normal saline) was injected into the thrombus at a rate of 2.5 ml per hour for 20 hours. During the thrombolysis period, the patient showed modest clinical improvement in terms of reduced pain, numbness, and improved limb temperature. A check angiogram conducted after the infusion showed an improved vasculature but with a significant amount of residual thrombus remaining in the SCA, BA, and beyond. Further thromboaspiration was carried out using an AXS Catalyst® 6 catheter (Stryker Corporation) inserted within the 7F long sheath, leading to the successful aspiration of most of the clot and revascularization up to the palmar arch (Figure 3).

**Figure 4:**
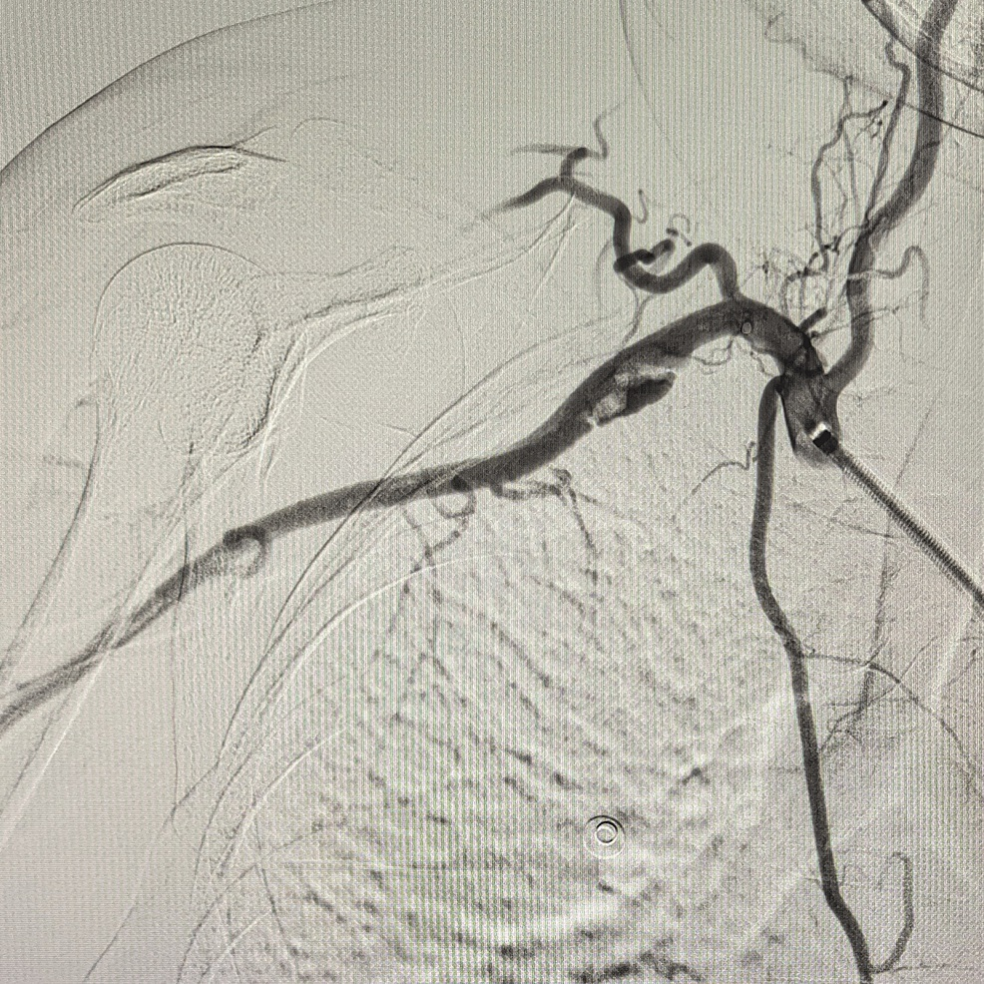
DSA of the right SCA showing a dissection with a focus of thrombus. DSA, digital subtraction angiogram; SCA, subclavian artery.

However, at the completion angiogram, a dissection was noticed at the SCA with a focus of thrombus. It was recognized that this dissection could lead to recurrence in the future, and a decision was taken to stent the lesion (Figure 4).

**Figure 5:**
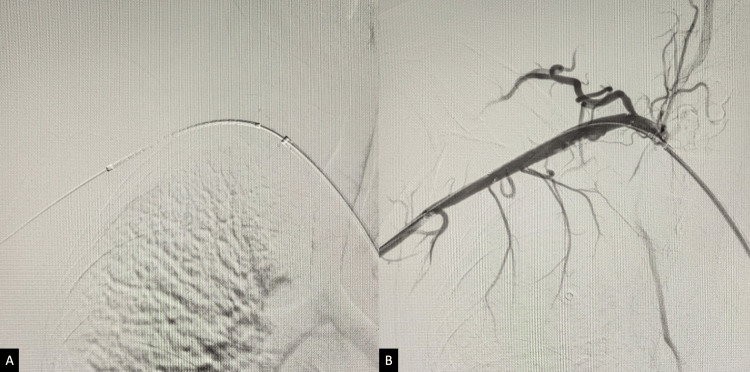
Fluoroscopy showing the placement of the stent-catheter assembly across the dissected segment prior to the deployment and expansion of the stent (A). Final check angiogram demonstrating a sufficient flow across the stent (B).

After the loading dose of antiplatelets (180 mg of ticagrelor and 150 mg of aspirin), a 7 x 60 mm self-expanding stent (E-Luminexx^TM^, C. R. Bard Inc., Tempe, Arizona, United States) was deployed into the dissected segment, completely covering it (Figure 5).

A final check angiogram demonstrated good contrast flow all the way to the palmar arch. Within the next 48 hours, the patient showed remarkable improvement, with pain nearly eradicated and with local warmth with a strong bounding pulse of the radial and ulnar arteries. The post-treatment sensorimotor functions were normal. There were no instances of bleeding or other periprocedural complications. She was discharged on a combination of ticagrelor (180 mg/day) and aspirin (75 mg/day). The patient’s sixth-month follow-up Doppler was normal, and she had no other clinical complaints.

## Discussion

Spontaneous SCAD without a history of prior trauma or angiographic procedures is a rare occurrence [3]. The exact pathogenesis of arterial dissection is unclear, but possible associating factors include trauma (even minimal trauma associated with sports), hypertension, vasculopathy, migraine, and drug abuse [5].

Unruptured arterial dissections have a relatively benign clinical course and outcome. Therefore, conservative management, such as antiplatelets, anticoagulation, or combination therapy, is typically recommended [6-9]. However, the natural history of this lesion remains poorly understood, and the optimal treatment has not been established. Some dissections undergo spontaneous reconstitution of the vascular lumen and heal effectively. However, some dissections can cause thromboembolic events that lead to ischemic sequelae and bleeding, resulting in significant morbidity and mortality [9].

SCAD can cause chest, back, and neck pain, a pale and cold limb with pulselessness, dizziness with nausea and vomiting, or visual disturbances [1,10-12]. Possible complications of SCAD include thrombosis, emboli to the head and neck or upper extremity, and ischemic sequelae in the affected organ, as well as pseudoaneurysm, intramural hemorrhage, or subclavian steal syndrome [3,5,13].

In our patient, there was no evidence of systemic arteriopathy, connective tissue disorders, or other contributing factors. The clinical presentation was critical limb ischemia of the right upper extremity, with no neurological complaints. Emergency revascularization was necessary due to the acute nature of this presentation to save the limb and prevent amputation. Conventional conservative management would not have yielded the required results on time.

Surgical repair is only recommended for symptomatic patients with lesions in accessible locations. However, the approach for preserving the artery is technically demanding, time-consuming, and associated with a higher risk of bleeding, local trauma, infection, anesthesia-related risks, and prolonged hospitalization and recovery compared to the minimally invasive nature of endovascular therapy [9,14].

If a SCAD leads to ischemia, endovascular treatment of the lesion produces good outcomes [5,10,15]. Stents, due to their design, provide a centrifugal force that apposes the dissected segment to the vessel wall to obliterate the false lumen and preserve the patency of the artery. Although data availability is limited due to the rarity of spontaneous dissections in this region, stenting for dissections in the carotid and vertebral arteries has been associated with good outcomes [9,16,17].

As observed in our case, timely revascularization yields an exceptional outcome. However, large-scale studies are needed to further validate this claim.

## Conclusions

Although SCAD is a rare condition, it can occur without the presence of noticeable comorbidities. Endovascular therapy is a safe and effective treatment modality without any significant drawbacks. Early recognition and prompt treatment are crucial for achieving the best possible outcomes.
